# Extracellular vesicle-cell adhesion molecules in tumours: biofunctions and clinical applications

**DOI:** 10.1186/s12964-023-01236-8

**Published:** 2023-09-21

**Authors:** Weikai Lin, Jianjun Fang, Shibo Wei, Guangpeng He, Jiaxing Liu, Xian Li, Xueqiang Peng, Dai Li, Shuo Yang, Xinyu Li, Liang Yang, Hangyu Li

**Affiliations:** 1 Department of General Surgery, The Fourth Affiliated Hospital, China Medical University, Shenyang, 110032 China; 2 Shenyang Clinical Medical Research Center for Diagnosis, Treatment and Health Management of Early Digestive Cancer, Shenyang, 110032 China

**Keywords:** Extracellular vesicles, Exosomes, Cell adhesion molecules, Tumour microenvironment, Clinical applications

## Abstract

Cell adhesion molecule (CAM) is an umbrella term for several families of molecules, including the cadherin family, integrin family, selectin family, immunoglobulin superfamily, and some currently unclassified adhesion molecules. Extracellular vesicles (EVs) are important information mediators in cell-to-cell communication. Recent evidence has confirmed that CAMs transported by EVs interact with recipient cells to influence EV distribution in vivo and regulate multiple cellular processes. This review focuses on the loading of CAMs onto EVs, the roles of CAMs in regulating EV distribution, and the known and possible mechanisms of these actions. Moreover, herein, we summarize the impacts of CAMs transported by EVs to the tumour microenvironment (TME) on the malignant behaviour of tumour cells (proliferation, metastasis, immune escape, and so on). In addition, from the standpoint of clinical applications, the significance and challenges of using of EV-CAMs in the diagnosis and therapy of tumours are discussed. Finally, considering recent advances in the understanding of EV-CAMs, we outline significant challenges in this field that require urgent attention to advance research and promote the clinical applications of EV-CAMs.

Video Abstract

**Supplementary Information:**

The online version contains supplementary material available at 10.1186/s12964-023-01236-8.

## Introduction

Cell adhesion molecules (CAMs) enable cellular adherence and interactions with the extracellular matrix (ECM) and other cells [1]. Depending on their structural characteristics, CAMs can be categorized into the cadherin family, integrin (ITG) family, selectin family, immunoglobulin superfamily, or a variety of adhesion molecule group that have not yet to be classified [2]. In addition to facilitating cell attachment, CAMs can transport external biochemical [3, 4] and transduce biomechanical signals [5, 6] to regulate cellular processes, such as cell shape, dynamics, proliferation, differentiation, and gene expression [7–11]. Dysregulation of CAM-mediated adhesion and signalling is critical to the pathogenesis of several illnesses, such as cancer, cardiovascular disease, muscular dystrophy, and haematologic disorders [12]. Aberrant expression of CAMs has been found in various tumours. CAMs are believed to be connected to tumorigenesis, remodelling of tumour and ECM cells, and tumour cell migration and metastasis, and resistance to anticancer therapy [13–17]. Increasing evidence suggests that CAMs function as cargos of extracellular vesicles (EVs) to participate in information transfer and affect the function of recipient cells [18, 19].

EVs are lipid bilayer-structured particles released by cells into the extracellular environment [20]. All classes of cells can release EVs [21–26]. The presence of EVs can be detected in various body fluids, including blood [27], breast milk [28], saliva [29], and urine [30]. EVs are usually classified into several categories, such as exosomes, microvesicles, and apoptotic bodies [20, 31, 32]. In this review, we outline the role played by CAMs as EV cargos, the in vivo distribution and uptake of these EVs and the impact of EV-promoted crosstalk among cells in the TME on tumorigenesis and tumour progression. In addition, we provide new ideas for basic research and clinical applications of EV-CAMs in tumour proliferation and metastasis.

### Biogenesis of EVs and CAMs loading onto EVs

#### Biogenesis of EVs

Based on their biogenetic pathways, EVs can be classified into several main classes, including exosomes, microvesicles, and apoptotic bodies.

#### Biogenesis of exosomes

Exosomes are EVs that have a diameter between 50 and 150 nm [20]. There are two primary pathways involved in the formation of exosomes: the endosomal sorting complex required for transport (ESCRT)-dependent pathway and the ESCRT-independent pathway [33]. Early endosomes, which are formed by invaginated plasma membrane budding, gradually mature into late endosomes. Subsequently, the inward budding of the late endosomal membrane forms intraluminal vesicles (ILVs), which are contained within multivesicular bodies (MVBs) [34]. ESCRT-0, ESCRT-I, and ESCRT-II help load cargos into the lumen of MVBs by deforming membrane structures and generating budding. ESCRT-III is recruited by ESCRT-II to cut bud necks and form ILVs in the MVB lumen. Auxiliary proteins such as VPS4, Snf7, and ALG-2 interacting protein X (ALIX) facilitate this process. In addition, some ESCRT-independent pathways controlled by ceramide [35], Rab31 [36], ADP ribosylation factor 6 (ARF6) and phospholipase D2 (PLD2) [37]. MVBs can be carried to lysosomes for degradation. Alternatively, MVBs can transport (mainly controlled by Rab GTPases), fuse with the plasma membrane (mainly controlled by SNARE complex) and release ILVs into the extracellular space, which results in the release of exosomes [33, 34]. Details are available in an article by Wollert [38] and a review by van Niel [31].

#### Biogenesis of microvesicles

Microvesicles (MVs) can range in size from 150 nm to more than 1,000 nm in diameter [20]. MVs are created through plasma membrane budding without the involvement of MVBs. Changes in membrane stress and modifications to membrane curvature contribute to localized membrane budding, which is facilitated by ATP-dependent aminophospholipid translocases and Ca2+-dependent scramblases [39]. MV release requires cytoskeleton phosphorylation and contraction. The GTPase RhoA is an important regulator of this process, causing activation of ROCK and LIMK and phosphorylation of cofilin [40].

#### Biogenesis of apoptotic bodies

Apoptotic bodies constitute the largest subpopulation in EVs in terms of volume, with sizes typically ranging from 1 µm to 5 µm in diameter [20]. Programmed cell death creates apoptotic protrusions from the plasma membrane that extrude into the cell matrix to form apoptotic bodies [41]. Intracellular cargos are transferred in apoptotic cells by microtubules [42]. Moreover, ROCK-I enhances membrane budding and apoptotic body release by triggering actin-myosin contraction [43]. Notably, that apoptotic bodies and MVs are produced through processes with some similarities, but the latter are characterized by organelle disruption and nuclear genomic fragments, while former are not [20].

EVs have been classified into exosomes, microvesicles, and apoptotic bodies, among others types, according to their different biogenetic pathways. However, identifying the pathway of origin of EVs remains very difficult due to the difficulty in obtaining a completely single, high-purity EV of a specific type via techniques and the lack of consensus about the specific markers for each EV subtype. Therefore, the most recent classification guidelines distinguish EVs by physical characteristics (e.g., size) [44]. Moreover, the early literature in the EV field lacked standardized terms or unified theories, and the origin and characterization of EVs were thus not adequately described in many studies [45]. Therefore, we use the generic term EVs instead of exosomes, microvesicles, and apoptotic bodies later in the review to avoid misleading readers.

#### CAMs loaded onto EVs

One significant method of intratumoral communication is the dispersion of CAM-loaded tumour-derived EVs (TDEs). A few CAMs, including some ITGs and tetraspanin (Tspan), can be used as markers in EVs according to relevant guidelines [44]. However, a large-scale proteomic analysis based on EVs showed significant differences in ITG expression between EVs of different cell origin [46]. This indicates that CAMs, as well as other cargos loaded onto EVs, exhibit significant heterogeneity, and only a few CAMs can also be used as markers to indicate the origin or function of a particular cell type [47]. Galectin-3 (Gal-3) mediates two important steps in EV biogenesis: endocytosis and cargo delivery to the membrane. After Gal-3 expression was knocked down, the number of TDEs carrying CAMs was considerably reduced, which then indirectly inhibited tumour cell metastasis and colonization [48].

To date, there are profound challenges confounding the idea of a uniform mechanism for loading CAMs onto various EVs because of the intrinsic heterogeneities of EVs, the variety of CAM species and the lack of studies on the loading of most CAMs. However, loading mechanisms among different kinds of CAMs, as well as other cargos, such as membrane proteins, may be the same [49, 50]. Some common mechanism can certainly influence CAM loading. Knocking down the ALIX gene, which promotes ILV formation, led to defects in vesicle release and transport, which depend on the MVB pathway, ultimately lowering the number of several types of CAMs on EVs [51]. In addition, the amount of CAMs, similar to that of other cargos, contributes to the loading process [52]. For example, ITG β3 carried by kidney-derived EVs; ITG α3 and α6 carried by EVs of colonic and ovarian cell origin, respectively; ITG β1 commonly found in benign breast EVs, and ITG α2 and ITG α3 carried in malignant breast EVs were positively correlated with ITGs carried by the EVs and the ITGs expressed by parental cells. This finding implied a direct correlation between the amount of ITGs in EVs and the amount of protein expressed on parental cells [46]. In contrast, under some conditions, a strong negative correlation has been found between CAM (e.g., ITG αv) expression on the cells and their levels on EVs [46]. This indicates the specific loading mechanisms for unique CAMs. Another good example is that ITG β1 is enriched in ARF6-regulated MVs in certain cell type but is rarely carried in exosomes [53, 54]. In contrast, Imjeti et al. found that SRC signalling was regulated upstream of ARF-6 to promote the loading of ITG β1 onto exosomes released from a different parental cell type [55]. However, details explaining these loading mechanisms are rare. Exploring the specific mechanisms of CAM loading onto EVs will greatly facilitate researchers' understanding of intratumoral communication.

### EV-CAMs facilitate EV interact with target cells

#### EV-CAMs regulate the distribution of EVs in vivo

EVs show the capability to attach to the extracellular matrix and engage with local recipient cells, or alternatively, they can be dispersed via the circulatory system and interact with distant cells, tissues, and organs [56, 57]. The differential spatial distribution of EVs is also influenced by the various levels of EV cargos and receptors on various target cells [58]. CAMs on the EV surface (which also includes integrins, tetraspanins, etc.) have been suggested to affect EV dispersion by mediating EV binding to differentially expressed ligands in specific cell types or organs [59–61]. For example, EVs carrying Tspan8 and ITG β4 preferentially bind to spleen, lung, and kidney tissues [60]; EVs expressing ITG α5β1 and αVβ3, which interact with fibronectin, exhibit preferential liver targeting [62]; EVs carrying ITG β3 aggregate in the brain [18]; and EVs-packaged ITG α5 targeting bone following osteoblast uptake of EVs [63]. During the circulation of EVs in vivo, these CAMs specifically bind to resident cells in organs, such as epithelial cells, endothelial cells, and phagocytes, to varying degrees [18, 64, 65]. The difference in the affinity of ligands for CAMs on receptor cells allows these cells to capture EVs carrying specific CAMs, resulting in EVs exhibiting a preference for specific tissues or organs. It should be noted that the examples provided in this article regarding EVs' targeting of organs and tissues are not exhaustive. Specific accumulations of EVs have also been observed in organs such as the gastrointestinal tract and the spine [66, 67]. However, it is premature to conclude that EV-CAMs are involved in these targeted accumulations in these organs at present.

In addition to the positive correlation between the expression of CAMs on EVs and the internalization of EVs by cells, it has been shown that CAMs are negative regulators of EV internalization. By inhibiting macrophage and monocyte phagocytosis of EVs, CD47, a widely expressed integrin-associated immunoglobulin superfamily protein, fine-tunes EV targeting to particular tissues and is used as a vehicle for drug delivery [68]. However, although EVs travel great distances through the vascular and lymphatic circulatory systems to affect particular tissues, how EVs enter the vascular system and travel across the endothelium are significant unaddressed problems in the field of EV research due to the lack of conclusive mechanistic studies [58]. Our current understanding of the mechanisms by which EVs pass the endothelial barrier is limited to evidence gathered in studies of the blood‒brain barrier and cannot be generalized to EVs movement through the systemic circulatory system. However, recent research based on CAMs has revealed that circulating tumour cells (CTCs) expressing CAMs are particularly effective in transendothelial migration and are capable of metastatic spread [7, 69]. Moreover, several CAMs have been associated with endothelial cell binding are expressed in similar amounts on CTCs and EVs and are crucial for the initiating the same adhesion-based movement patterns of cells [70]. Therefore, considering the results of some of these studies, we speculated that the pattern of EVs and CTCs crossing the endothelial barrier in vivo and those circulating in the blood is the same, i.e., to cross both barriers, EVs equally rely on the adhesion of CAMs on their surface and that of CTCs to ligands on endothelial cells. A recent study by Shima Ghoroghi et al. supports this hypothesis. They found that EVs expressing CD146 (MCAM) exhibited lung-targeting ability. In contrast, when CD146 was expressed at low levels on EVs, few EVs attached to the endothelial wall, impairing EV migration to the lung [64]. Similarly, CD146 expression is also found on the surface of tumour cells and peripheral blood cells, and its presence promotes cell extravasation, metastasis, and homing [71–73]. However, despite these findings, questions regarding the precise role of CAMs in EV metastasis remain unanswered. For example, it remains unclear whether CAM ligands on vascular endothelial cells differ in various organs and tissues. Additionally, the impact of CAMs on EV infiltration and mobility requires further investigation. Addressing these concerns will provide a more comprehensive understanding of how CAMs influence the metastatic behaviour of EVs.

#### The interaction of EV-CAMs with recipient cells

The interaction between EVs and receptor cells, which allows EVs to transfer proteins, lipids, and RNA cargos to cells and thus affect the receptor cell phenotype, is crucial to understanding how EVs functions in physiological and pathological processes [58]. EVs can fuse with the plasma membrane directly or be internalized into the cell when they fuse with endosomal membranes via a variety of specific and nonspecific pathways, including the clathrin/caveolin-mediated endocytosis, macropinocytosis, and phagocytosis pathways, and lipid raft-regulated uptake to transmit information [74]. Integrin is a CAM involved in the endocytosis of EVs. The interaction between ITGs and ligand proteins can be predicted by differences in the C-terminal sequence of an ITG. Heparan sulfate proteoglycan (HSPG)-modified proteins on the cell surface play roles in the recognition of EVs carrying ITG β3, and these EVs promote DYNAMIN-dependent endocytosis, which is initiated by FAK-dependent phosphorylation; capture of EVs by HSPG-modified proteins and DYNAMIN-driven endocytosis work in concert to increase uptake of ITG β3-expressing EVs [75]. Meanwhile, the inactive integrin conformation in EVs can be activated by talin, increasing the affinity of the integrin for the ligand [76]. Additionally, the efficiency of EV absorption is regulated by the coexpression of tetraspanins, such as CD81 or Tspan8 in complex with the integrin, on the plasma membrane [60, 77]. Moreover, EV absorption has been linked to some CAMs that are localized on the EV surface, including CD9, CD11a (a component of lymphocyte function-associated antigen-1 (LFA-1)), and several integrin family proteins [18, 78]. However, further research is required to fully understand how EV surface ligands function because of their intricacy.

**Fig. 1:**
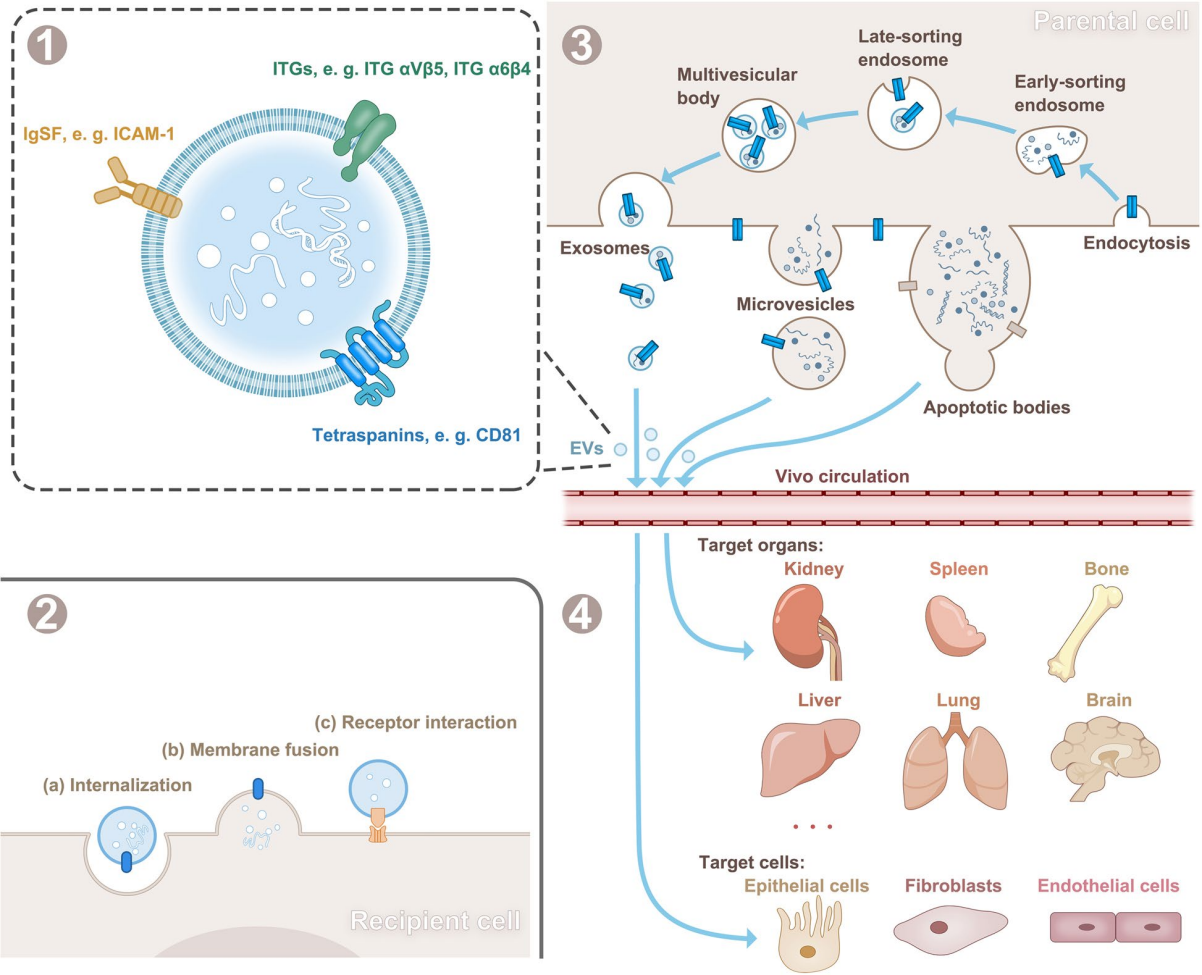
EVs carrying CAMs from parental cells to recipient cells. (1) CAMs transported by EVs; (2) released EVs can deliver information after internalization, fusion with the cell membrane, or binding to receptors on the surface of recipient cells; (3) the biogenesis and release of EVs into the circulatory system in vivo; and (4) EVs can interact with specific organs and cells

In addition to being internalized into recipient cells, EVs can transmit messages by attaching to ligands on target cells through the action of CAMs. It has been reported that the binding of intercellular adhesion molecule-1 (ICAM-1) on EVs and LFA-1 on T cells is accompanied by the interaction of PD-L1 coexpressed on EVs with PD-1 on T cells to inhibit T-cell activation and proliferation [79]. To control cellular activity, this method of binding frequently necessitates the activation of signalling pathways downstream of ligand binding. In conclusion, understanding the various modes of EV delivery adds to our knowledge of intercellular signalling (Fig. 1).

### The role of EV-CAMs in the tumour microenvironment

**Fig. 2:**
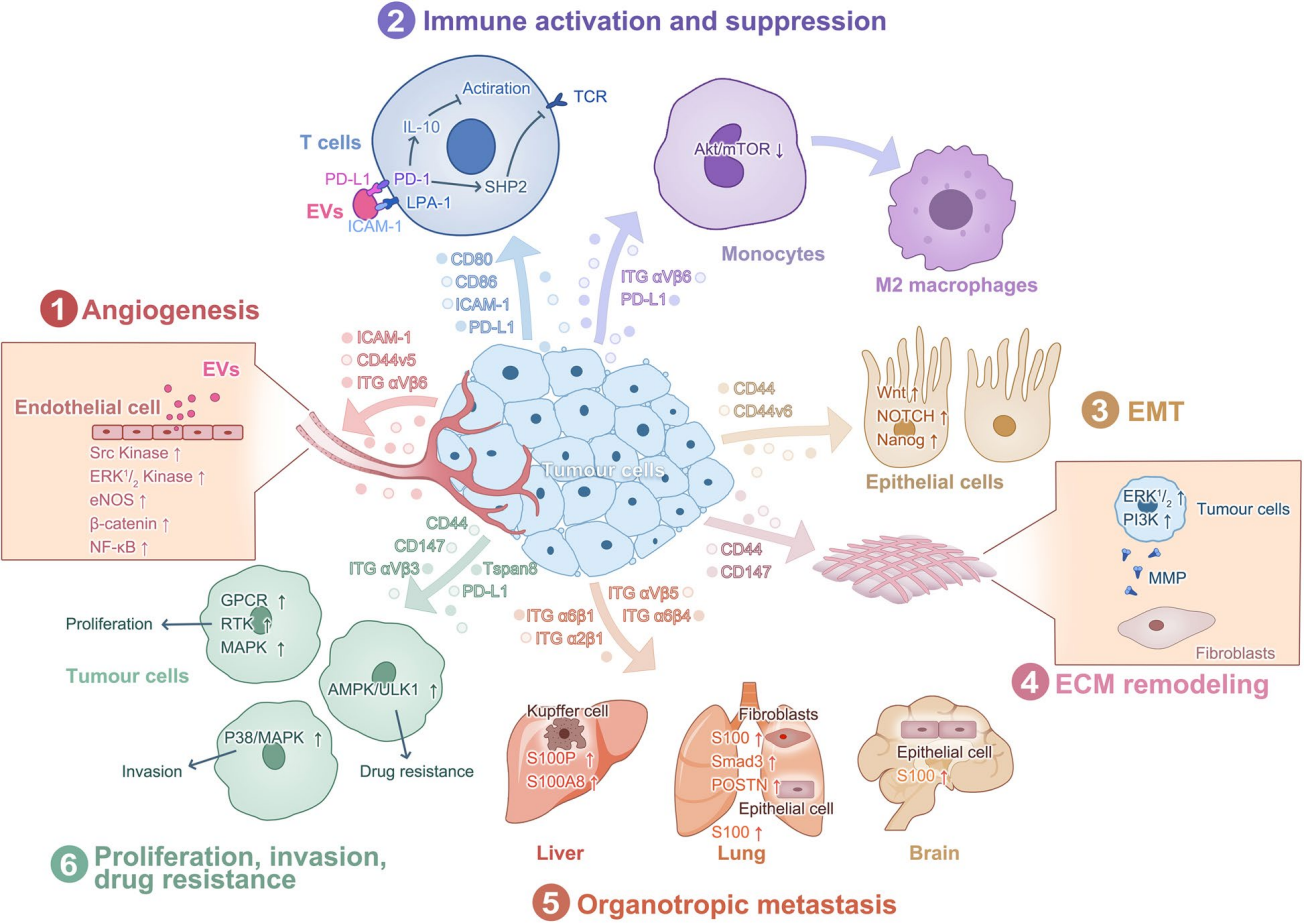
Signal exchange in TME via CAMs transported by EVs

Tumorigenesis and proliferation are not solely driven by genetic and epigenetic alterations within tumour cells. Researchers, including Hanahan and Weinberg, have shown that these processes are also influenced by the acquisition of several hallmarks of tumour cells in the TME [80–82]. The TME comprises various cell types, vascular and lymphatic networks, extracellular matrix, and biomolecules, influencing tumour growth, invasion, and metastasis [81, 83]. In addition to influencing EV movement and absorption in vivo, CAM functions as cargo carried by EVs and exhibits the ability to activate signalling pathways that exert a significant impact on the TME and modulate tumour cell activity. For example, EV-CAMs function a modulator of immune cells, regulating the activity, function and differentiation state of immune cells to influence tumour development and immune escape [84]. In addition, EV-CAMs derived from tumour cells or tumour-associated cells in the TME mediate various biological functions, including but not limited to promoting angiogenesis, ECM remodelling, the epithelial-mesenchymal transition (EMT), and the formation of premetastatic niches (PMNs) [18, 85–87]. To encourage tumour invasion and metastasis, several systems function together [18, 79, 85–88]. The intricate relationship between EV-CAMs and the TME is reviewed in this article, and representative not all-inclusive instances are described (Fig. 2, Table 1).

**Table 1:** Functions of CAMs carried by EVs in the tumour microenvironment

CAM family member	Introduction	CAMs carried by EVs	Donor cells	Recipient cells	Functions in TME	Reference
The immunoglobulin superfamily	The immunoglobulin superfamily (IgSF) is one of the largest and most diverse families of proteins. The common feature of IgSF members is an immunoglobulin domain, and most members are Type I transmembrane proteins with extracellular domains (carrying Ig domains), transmembrane domains and cytoplasmic tails	ICAM-1	Nasopharyngeal carcinoma cells	Endothelial cells	Enhances angiogenic capability by altering signalling pathways, including the src kinase, ERK1/2 kinase, and eNOS pathways, in endothelial cells	[85]
		PD-L1	Melanoma cells, lung cancer cells and colon cancer cells	T cells	Recruits SHP2 to inhibit the downstream transduction pathway triggered by a TCR and leads to T-cell dysfunction	[89]
			Breast cancer cells	Monocytes	Inhibits serine-threonine kinase AKT/mTOR signalling in macrophages, mediating macrophage differentiation towards the M2 phenotype	[90]
			Glioblastoma stem cell	Glioblastoma cells	Activates the AMPK/ULK1 signaling cascade to induce protective autophagy, resulting in drug resistance	[91]
			Myeloid-derived suppressor cells	T cell	Suppresses the antitumour immune response of CD8^+^ T cells	[92]
		EMMPRIN	Peritoneal mesothelial cells	Gastric cancer cells	Regulates MMP release through the ERK1/2 and PI3K signalling pathways	[86]
			Lung carcinoma cells, breast cancer cells	Fibroblasts	Promotes the production of MMP	[93, 94]
			Breast cancer cells	Breast cancer cells	Increases cell invasion by activating the p38/MAPK signalling cascade	[95]
The integrin family	Integrins are heterodimers consisting of two subunits, the alpha and beta subunits. These subunits each harbour transmembrane structures and extracellular structural domains. the combinatorial diversity of the 18 alpha and 8 beta subunits results in multiple isoforms in the integrin family and confers specific binding affinity for different ligands	ITG αVβ3	Prostate cancer cells	Prostate cancer cells	Induces the differentiation of receptor cells into neuroendocrine NEPrCa cells	[96]
			M2-like macrophage	Non-small cell lung cancer	Activates the FAK/p-FAK signalling pathway to promote the progression tumour cells	[97]
		ITG αVβ6	Prostate cancer cells	Endothelial cells	Drives vascular endothelial cell migration to initiate the angiogenic programme	[98]
			Prostate cancer cells	Monocytes	Promotes monocyte M2 polarization	[99]
		ITG αVβ5	Pancreatic cancer cells	Liver Kupffer cells	Activates the expression of S100P and S100A8 to promote the formation of a liver premetastatic niche	[18]
		ITG β4	Triple-negative breast cancer	Cancer-associated fibroblasts	Triggers glycolysis in CAFs	[100]
		ITG α5β1	Cancer-associated fibroblasts	Pancreatic cancer cells	Supports the survival of tumour cells by activating the NetG 1/NGL-1 axis	[101]
		ITG α6β1	Breast cancer cells	Lung fibroblasts and epithelial cells	Activates the expression of S100 to promote the formation of a lung premetastatic niche	[18]
		ITG α6β4	Breast cancer cells	Lung fibroblasts and epithelial cells	Activates the expression of S100 to promote the formation of a lung premetastatic niche	[18]
		ITG α2β1	Salivary adenoid cystic carcinoma-associated fibroblasts	Lung fibroblasts	Induces the expression of phosphorylated Smad3 and POSTN to promote the formation of a lung premetastatic niche	[102]
The cadherin family	Cadherin is named because of its dependence on calcium ions (Ca^2+^) for its action. There are several subtypes, including classical cadherin type I proteins (e.g., E-cadherin, N-cadherin, etc.) and classical cadherin type II proteins (e.g., desmosomal cadherin, VE-cadherin and protocadherin, etc.). Almost all cadherin are transmembrane proteins consisting of three components, namely, an extracellular cadherin domain, a single-transmembrane domain and a cytoplasmic domain	E-cadherin	Ovarian cancer cells	Endothelial cells	Dimerizes with VE-cadherin on endothelial cells and transduces the activation of β-catenin and NF-κB signalling	[103]
Other CAMs	Play a role in cell adhesion	CD44v5	Nasopharyngeal carcinoma cells	Endothelial cells	Stimulates angiogenesis by promoting the proliferation and migration of endothelial cells as well as controlling the adhesion between endothelial cells and the extracellular matrix	[85, 104]
		CD44v6	Pancreatic cancer cells	Pancreatic cancer cells	Activates the Wnt, NOTCH, and Nanog signalling pathways to mediate the EMT	[105]
					Activates GPCR, RTK, MAPK pathway to promote tumour proliferation	
					Promotes the overexpression of an ABC drug transporter protein, conferring drug resistance on tumour cells	
		CD44	Epithelial ovarian cancer cells	Peritoneal mesothelial cells	Promotes the EMT	[87]
					Promotes MMP9 secretion	
			Ovarian cancer cells	Ovarian cancer cells	Reprogrammes less-aggressive cancer cells into more aggressive cancer cells	[88]

TME alterations are regulated by tumour-derived EVs, which communicate with autologous cancer cells or stromal cells to influence tumour progression. CAMs transported by EVs are involved in a variety of biological processes, including angiogenesis, immune activation and suppression, the epithelial-mesenchymal transition, and ECM remodelling. The factors that cause tumour cell invasiveness, sustained proliferation, and drug resistance can be transmitted between different tumour cell subpopulations via EVs. Moreover, CAMs transported by EVs are important determinants of PMN location and regulate their formation. Multiple mechanisms function in concert to promote tumour proliferation, invasion, and metastasis.

#### Angiogenesis

A crucial step in tumour progression is angiogenesis, through which tumours obtain O_2_ and nutrient support while expelling CO_2_ and metabolic waste [81]. Angiogenesis involves the proliferation and migration of endothelial cells, extracellular matrix remodelling, and the development of angiogenic tubes [106]. Recent research has indicated that the actions of EV-CAMs modifies endothelial cell function and promotes angiogenesis in malignancies. Chan's team discovered that EVs derived from nasopharyngeal carcinoma (NPC) cells exhibited high levels of ICAM-1 and CD44 variant 5 (CD44v5) expression. These EVs exerted an effect on several signalling pathways, including the src kinase, ERK1/2 kinase, and endothelial nitric oxide synthase (eNOS) pathways, which are related to endothelial cell adhesion, migration, and proliferation and ultimately drove endothelial cell angiogenesis [85]. Similarly, another study showed that integrin αvβ6 in prostate cancer (PrCa)-derived EVs drove vascular endothelial cell migration to initiate the angiogenic programme after being internalized into recipient cells. These EVs also drove the downregulation of pSTAT1 and the activation of TGF-β1, blocking the STAT1 signalling pathway to promote angiogenesis [98]. In the future, targeting proangiogenic EV-CAMs in a new treatment approach to solid tumours may be possible because EV-CAMs can drive angiogenesis, which is a hallmark of tumours.

#### Immunity

EV-CAMs are of great importance to the maintenance of the immune network. During antigen recognition, peptide-MHC presented after antigen-presenting cell (APC) uptake is needed to activate naive T cells, and it is facilitated by EV delivery when APCs do not directly engage T cells [107]; this process also requires coexpression of ICAM-1 and B7-1 (CD80)/B7-2 (CD86) on EVs [108, 109]. These proteins are members of the immunoglobulin superfamily; ICAM-1 is critical for cell adhesion to LFA-1, while CD80/86 attaches to CD28 on T cells to induce a developmental secondary signal, which is crucial for the activation of naive T cells. However, in studying this process, researchers frequently focus on the APCs that can deliver EVs to T cells while ignoring the presentation of the substances between APCs and T cells, which is actually a bidirectional process. For instance, integrin LFA-1-containing EVs produced by T cells can be recruited to dendritic cells (DCs). These EVs induce apoptosis in DCs via the Fas/FasL pathway to mediate T-cell regulation of DC immune function [110, 111]. The transmission of these immunological signals is crucial for the development and maintenance of antitumour immunity.

Immune escape is a crucial characteristics of malignancies [81]. According to studies performed thus far, EV-CAMs delivery between tumour cells and immune cells contributes to the formation of an immunosuppressive TME that promotes tumour growth. Programmed cell death-ligand 1 (PD-L1) is not only expressed on the cell surface but can also be loaded onto EVs under the regulatory action of ALIX [51]. Recently, Zhang et al. discovered that ICAM-1 and PD-L1 were coexpressed on TDEs, and the expression of both proteins was upregulated by IFN-γ. ICAM-1 is a positive regulatory protein that mediates the involvement of PD-L1 on immunosuppressive TDEs, which is important because the interaction between PD-L1 and PD-1 is weak, and the high affinity of ICAM-1 for the ligand LFA-1 triggers effective EV–cell contact [79]. Recent studies have shown that PD-L1, when in contact with the ligand programmed death protein-1 (PD-1), induces the expression of interleukin 10 (IL-10), a suppressive immune response regulator [112], as well as the aggregation of PD-1 with the T-cell receptor (TCR), which recruits Src homology 2 domain-containing tyrosine phosphatase 2 (SHP2), inhibiting the downstream transduction pathway activated by the TCR and leading to T-cell dysfunction [89]. This outcome is particularly notable when activated CD8^+^ T cells are involved because these cells secrete IFN-γ to inversely regulate the expression of ICAM-1 and PD-L1 on TDEs [79], which is a reason for that TDEs can recognize and block the killing effect of activated CD8^+^ T cells. Additionally, the binding of ICAM-1 in EVs to T cells inhibits T-cell migration and aggregation mediated by the LFA-1 and ICAM-1 interactions between T cells and endothelial cells [113]. Future studies are required to gain a better understanding of EV-CAMs function because of the intricate mechanisms involved in immune modulation mediated by EV-CAMs. These studies will not only increase our knowledge of the immunosuppressive TME but also help in the development new targets for tumour immunotherapy.

In addition to suppressing the ability of immune cells to kill tumours, EV-CAMs promote the phenotypic polarization of immune cells towards a protumorigenic phenotype, which contributes to the generation of an immunosuppressive TME. For instance, Lu et al. demonstrated that ITG αvβ6 expressed on TDEs and transferred to monocytes suppressed the expression of STAT1 and MX1/2 in the monocytes, enabling them to differentiate towards the tumour-promoting M2 phenotype not the M1 phenotype [99]. In addition, Li et al. discovered that tumour-derived PD-L1^+^ EVs inhibited serine-threonine kinase AKT/mammalian target of rapamycin (mTOR) signalling on macrophages, mediating macrophage differentiation towards the M2 phenotype to accelerate triple-negative breast cancer progression [90]. The aforementioned data indicate that the mechanisms underlying tumour immunity are complex, and the same CAMs loaded onto EVs may regulate the immune functions of different immune cells. To understand the intricate tumour immune network, further research is needed.

#### Tumour cell invasion and metastasis

##### The epithelial–mesenchymal transition

The EMT is a cell biological programme that regulates how aggressive tumour cells behave around the edges of solid tumours [82]. Epithelial cells lose their characteristic apical-basal polarity, actin cytoskeletal arrangement, intercellular adhesion junction, and other structures and functions during EMT, resulting in a more migratory and invasive mesenchymal cell phenotype. [114–116]. We believe that TDEs carrying CAMs are among of the triggers that initiate the EMT in the TME. The results of an analysis of EMT markers and cell morphology showed that epithelial ovarian cancer (EOC)-derived EVs promoted the EMT independent of TGFβ signalling by transferring CD44^+^ EVs to human peritoneal mesothelial cells (HPMCs) [87]. However, TGFβ has frequently been considered a key factor involved in the EMT in various studies [117]. Moreover, Wang et al. also found that the CD44v6^+^EV-mediated EMT was associated with signalling stimulation of the Wnt, NOTCH, and Nanog pathways [105]. Notably, CD44^+^ EVs facilitate the development of the EMT, and conversely, the EMT can boost the secretion of CD44^+^ EVs by primary mesothelial cells [118]. However, whether this mutual promotion of the EMT and EV release is synergistic or whether it is a result of a positive feedback loop that affects tumour progression remains unclear.

##### Extracellular matrix remodelling

The ECM is a three-dimensional noncellular structure that is in intimate contact with cells and not only supports a tissue structurally but also harbours components that interact with cell ligands to mediate regulatory signalling [119]. In addition, the ECM is a highly dynamic structure that is continually changing. The interaction of cancer cells with the ECM in tumours alters the original biochemical, structural, and biomechanical properties of the ECM, facilitating tumour growth, invasion, and metastasis [119, 120].

According to recent research, EV-CAMs can trigger the release of matrix metalloproteinase (MMP), which degrades different ECM protein fractions, disrupting the histological barriers that prevent tumour spread. Extracellular matrix metalloproteinase inducer (EMMPRIN) can be expressed in mesothelial cells (MCs) in the TME, and can be delivered via EVs that are taken up by tumour cells after binding to EMMPRIN receptors on cancer cell membranes, thereby regulating MMP release in the cancer cells through the ERK1/2 and PI3K signalling pathways [86]. Similarly, tumour cells can release EVs expressing EMMPRIN, which promotes the production of MMP in adjacent fibroblasts [86, 93, 94]. The release of stored growth factors due to ECM degradation caused by MMPs activates intracellular signalling and contributes to tumour cell invasion [121]. Therefore, it is believed that EMMPRIN expression on the surface of tumour cells is linked to an aggressive cancer phenotype. In addition, CD44 is also a CAM-enriched TDEs [88]. Nakamura et al. discovered that ovarian cancer-derived EVs transfer CD44 into HPMCs, induce HPMC reprogramming, and promote the secretion of MMP9 to remodel the ECM [87].

In addition to the direct regulatory aforementioned effects of CAMs, CAMs carried by EVs can also facilitate the regulatory effects of other non-CAM biomolecules carried by EVs on receptor cells because of the adhesion mediated by CAMs, which can bind to many protein ligands in the ECM. Wei Mu et al. found that CAMs transported by pancreatic cancer-derived EVs with high affinity for the ECM effectively enhanced the ability of proteases carried by EVs, such as MMP7, MMP9, and ADAM17, to promote degradation of the ECM and procollagen maturation [122]. Additionally, the binding and uptake of these TDEs stimulate the release of ECM chemokines, which induce the proliferation and anti-apoptotic transformation of stromal cells. This outcome promotes the recruitment of endothelial cells and the development of myofibroblasts, which increase the invasiveness of these endothelial cells and fibroblasts in the TME [122]. Along with contributing to the loss of ECM suitability as a histological barrier, EV-CAMs participate in remodelling the ECM to release cellular molecules that can be used to control the activity of tumour cells and thus modulate tumour cell activity. Tumour cell motility and invasiveness are supported by the combined effects of changes in the ECM and tumour cells.

##### EV-CAMs promote tumour premetastatic niches development

One of the main causes of death in people with tumours is the metastatic progression of the tumours [123]. The traditional "seed-and-soil" theory, put forth by Stephen Paget in 1889, states that a proper microenvironment is the "soil" for the growth of tumour cells [124]. The initial "seed-and-soil" theory was later supported by researchers who proposed the concept of a PMN [125]; this theory suggests that tumour cells secrete signalling factors prior to metastasizing, targeting distant tissue sites in other organs that then undergo a sequence of changes to produce an ecological niche that permits tumour growth. According to recent studies, certain tumour cells may preferentially metastasize to a particular organ, not metastasize in a random way [123].

Studies have shown that TDEs are important mediators, playing dual roles for determining the location of PMN formation and regulating the formation process (regulation of vascular leakiness, recruitment of bone marrow-derived myeloid cells, etc.) [18, 126]. Studies have shown that through both roles, TDEs rely on CAMs. By analysing the TDE proteome of several tumour models, Hoshino et al. identified integrins as the most representative CAM family that displayed organ-specific metastatic tendencies and showed elevated the expression of S100 genes in target cells to promote PMN formation [18]. Specifically, pancreatic cancer-derived EVs carrying ITG αvβ5 preferentially attached to Kupffer cells, showed regulatory liver tropism, and activated S100P and S100A8, promoting PMN formation in the liver [18]. Additionally, TDEs carrying ITGα6β4 and ITGα6β1 preferentially bound fibroblasts and epithelial cells in the lung, activating the Src-S100A4 axis and S100A6, S100A10, S100A11, and S100A13 to promote PMN development and thus guide tumour cells to the lung, where they created metastases, while the expression of ITG β3 on EVs caused TDEs to accumulate in the brain [18]. In addition to TDEs, CAF-derived EVs can promote PMN development. CAFs in salivary adenoid cystic carcinoma secrete EVs carrying ITG α2β1, which target lung fibroblasts (LFs) and induce the increased expression of phosphorylated Smad3 (p-Smad3) and POSTN [102]. These EVs activated the AKT and STAT3 signalling pathways, attracting myeloid-derived suppressor cells (MDSCs) and forming PMNs in the lung [127]. In addition, other researchers have identified certain CAMs that promoted PMN formation, although they did not induce significant organotropism. For example, in ovarian cancer, sE-cad carried by EVs dimerized with VE-cadherin on endothelial cells, thereby triggering the activation of β-catenin and NF-κB signalling. This activation mechanism promoted the movement of endothelial cells and disrupted the integrity of the endothelial barrier. Thus, this alteration affected the permeability of endothelial cells, leading to vascular leakage and facilitating tumour propagation [103]. Additionally, in colorectal cancer, ITGBL 1-enriched TDEs stimulate the TNFAIP 3-mediated NF-κB signalling pathway to activate distal fibroblasts. As a result, activated fibroblasts produced high levels of proinflammatory cytokines to promote PMN formation [128].

However, the formation of PMNs is a complex phenomenon that requires the cooperative effects of multiple factors in TDEs [129], and the action of a single CAM is insufficient to induce this process. For example, Xie et al. discovered that CD44v6 in shuttled by TDEs needed the action of C1QBP to promote PMN formation, and CD44v6 alone was necessary but insufficient for the activation of hepatic satellite cells [130]. Ghoroghi et al. showed that immunoglobulin CD146 on EVs in metastatic breast cancer enhanced the formation of pulmonary PMNs, although more attention was placed on its guiding role [64]. In summary, in addition to focusing on CAM capacity to promote distinct alterations, such as proinflammatory genes in the TME as well as induce vascular leakiness, researchers cannot ignore the indirect effects of CAMs, which direct TDE colonization and uptake to promote PMN formation.

#### Other malignant biological behaviours induced by EV-CAMs

Crosstalk among EV-CAMs and TME components mediates the transmission of tumorigenic signalling, such as signals involved in tumour cell invasiveness, sustained proliferative capacity, and drug resistance.

The invasion − metastasis cascade, a multistep process that mediates cancer cell spread, is initiated after local tumour cell invasion [81]. In addition to the aforementioned EV-CAM-induced ECM remodelling, which creates a favourable environment for tumour cell invasion, EV-CAMs alter the activity of tumour cells to enhance their intrinsic invasive properties [70, 122]. For example, EVs from highly invasive ovarian cancer cell lines that characteristically overexpress CD44 can reprogramme ovarian cancer cells with low aggressiveness that have engulfed EVs into cells with a more aggressive phenotype [88]. Moreover, EMMPRIN carried by TDEs, which researchers have previously found to exhibit MMP stimulatory properties, can increase the cell invasion rate by activating the p38/MAPK signalling cascade in a non-MMP-dependent manner [95]. EV-CAMs can influence tumour cells to become increasingly aggressive, as this paper has explained. Nevertheless, researchers must understand that tumour cell invasion and metastasis cannot be accomplished by a single tumour cell type; in addition to the invasive capacity of the tumour cells themselves, tumour cell invasion and metastasis depend on complex signalling exchanges with the surrounding microenvironment, which necessitates further study.

Sustained proliferation is the most fundamental property of tumours [81]. In addition to abnormal proliferation of tumour cells caused by mutations in intracellular genes and disruption of feedback mechanisms, which promote abnormal growth [81], cells can maintain proliferation-related signalling through the delivery of foreign EV-CAMs [105]. In prostate cancer cells, integrin αvβ3 carried by TDEs plays a clear role in inducing the differentiation of receptor cells into neuroendocrine PrCa (NEPrCa) cells; this cell subtype expresses neuron-specific proteins and can activate -tumour-promoting pathways independent of androgen receptor (AR) action [96]. TDEs have been shown to transfer CD44v6 and Tspan8 into noncancer-initiating cells to promote pancreatic cancer tumorigenesis. For example, CD44v6 binds and activates G protein-coupled receptors (GPCRs), receptor tyrosine kinases (RTKs) such as EphA4 and Met, and the MAPK pathway [105], and it enhances Wnt signalling associated with LRP6 [105, 131], which increases tumour cell activation signalling. Tspan8 endows receptor cells with resistance to apoptosis via the activation of the PI3K/AKT pathway [105], diminishing the attenuating role of apoptosis in cancer development and indirectly promoting tumour proliferation.

Tumour cells drug resistance poses a significant challenge, rendering traditional chemotherapy, targeted therapy, and immunotherapy ineffective during treatment. EV-CAMs have been shown to contribute to the alteration of tumour cells phenotype, leading to treatment resistance. Studying glioblastoma (GBM) cells treated with the anticancer medication temozolomide (TMZ), Zheng et al. discovered that PD-L1 + EVs generated by glioblastoma stem cells (GSCs) may activate the AMPK/ULK1 signaling cascade to induce protective autophagy. This promotes the expression of Ki67 protein and tumor cell proliferation, resulting in resistance of GBM to TMZ [91]. Additionally, there is proof that TDEs expressing CD44v6 promote the overexpression of an ATP-binding cassette drug transporter protein via PI3K/Akt pathway activation, endowing tumour cells with drug resistance [105]. In addition to encoding a phenotype for tumour cell drug resistance, this study showed the potential for CAMs to induce the acquisition of this phenotype by drug-sensitive cells receiving signals from drug-resistant tumour cells. These studies might not only explain the poor treatment efficacy for some tumours but also represent a future breakthrough in the fight against tumour cell drug resistance.

#### Stroma-derived EV-CAMs in the TME

In addition to originating from tumours and regulating their own malignant biological behaviour, as described above, EV-CAMs are also involved in the communication between malignant and nonmalignant compartments of tumours (including but not limited to CAFs, tumour-infiltrating lymphocytes (TILs), tumour-associated macrophages (TAMs)), leading to the spatiotemporal evolution of intratumoral heterogeneity [132]. For example, triple-negative breast cancer (TNBC) cells stimulate tumour growth and angiogenesis by regulating CAF glycolysis rates through EVs that deliver the ITGB4 protein to the CAFs [100]; in contrast, ITG α5β1-enriched EVs from CAFs maintain the survival of pancreatic cancer cells cells in a state of nutrient deprivation by activating the NetG 1/NGL-1 axis [101]. Similarly, EVs carrying PD-L1 induced M2 polarization in macrophages [133], and EVs from M2-like macrophages could also activated the FAK/p-FAK signalling pathway by delivering ITG αVβ3 to promote the progression of non-small cell lung cancer [97]. Nonmalignant compartments also interact with each other through EV-CAM-mediated communication (for example, EVs from MDSCs carrying PD-L1 effectively suppressed the immune response of CD8^+^ T cells [92]), but studies of this phenomenon are rare.

### The roles of EV-CAMs in tumour diagnosis and therapy

#### EV-CAMs as diagnostic biomarkers

Early tumour detection is vital for prolonging patient survival, but traditional imaging results and analysis of surgical tissue biopsy samples cannot provide a comprehensive, accurate, or timely picture of the overall status of tumours [134–136]. In recent years, as our understanding of precision medicine has increased, liquid biopsy has emerged as a noninvasive, repeatable, and real-time approach for acquiring tumour specimen for analysis and identification [137–140]. EVs, in particular, are considered ideal biomarkers for determining tumour dynamics, as they transport biological cargos from cells of different tumour origins and are linked to malignant cell behaviour. Pertinent clinical studies based on liquid biopsy samples are underway globally [136]. Some EV-CAMs are thought to be useful for predicting tumour development and prognosis since they are specifically expressed in patients with distinct malignancies. For instance, individuals with head and neck squamous cell carcinoma and whose cells produce CD44v3^+^ EVs tend to present with higher amounts of immunosuppressive proteins. Additionally, higher tumour activity, more advanced disease stage, and lymph node metastasis are linked to CD44v3^+^ EVs [141]. Another study of EV-CAMs showed that the ITG α6A splice variant in EVs can be used for monitoring early recurrence in patients with pancreatic ductal adenocarcinoma [142]. Additionally, PD-L1 carried by EVs is thought to act as a biomarker for a number of tumour types, showing the capacity to be used to predict disease activity and tumour development and evaluate responses to immunotherapy [143–145]. These CAMs are transported by EVs and can be utilized as potential tumour markers; regrettably, most of the research in this field is currently restricted to the laboratory, and validation via large clinical trials is lacking.

#### Engineered EV-CAMs as tumour therapy tools

Due to low in vivo stability, delivery inaccuracy, and limited capacity to cross biological barriers and enter the circulatory system, chemotherapeutic drugs used in conventional oncology treatment need to be replaced with new drugs and drug delivery systems, which have has recently garnered the attention of many researchers [146]. The ability to deliver pharmaceuticals to designated sites safely, accurately, and effectively is the hallmark of an ideal drug delivery system, as such a system increases the effectiveness of drug utilization while lowering costs and dangerous side effects [146, 147]. Recent research has shown that due to their excellent safety profile and natural targeting abilities, EVs are regarded as novel drug delivery tools with significant translational potential [148]. However, EVs are thought to be cleared quickly via phagocytosis by macrophages in organs such as the liver and spleen, resulting in a half-life of only a few minutes to one-half hour in plasma [149–152], which may affect their stability as drug carriers. Modifying therapeutic EVs to increase their circulation times may increase the time for them to reach target tissues, increase the amount of drug transferred, and ultimately increase their therapeutic efficacy. Several cell surface CAMs, such as CD44 and PECAM-1, have been identified with antiphagocytic functions against macrophages, have also been considered candidates for modifying EVs and thus increase their circulation half-life [153]. In addition, changing the biodistribution of EVs is an important strategy to ensure that they act on target tissues [154]. As previously mentioned in this paper, the expression of specific CAMs carried by EVs confer EVs with the ability to target specific tissues. Engineering alterations and modifications based on this capacity may enable the drug delivery function of EVs [155]. In this section, we review how modified EV-CAMs are engineered and used for targeted tumour therapy.

Li et al. used Doxil-loaded TDEs to target tumours at the tissue of origin. They discovered that TDEs were more suitable to be carriers for antitumour drug delivery than other types of EVs because they were more likely to return to the original tumour tissue following systemic injection, perhaps due to the expression of certain integrins on the TDEs [156]. This result suggested a new approach for creating therapeutic vectors that are specifically targeted to sites of tumorigenesis. Based on EVs obtained from the urine of prostate cancer patients who expressed the urological cancer antigens E-cadherin and CD47, Pan et al. created a therapeutic nanoplatform packed with Fe_3_O_4_ and the chemotherapy drug doxorubicin (DOX). They were able to enhance the enrichment and uptake efficiency of nanocarriers at tumour sites by taking advantage of the low uptake of CD47 by macrophages and the homologous targeting ability between EVs and tumour cells. This strategy resulted in significant cytotoxic effects that inhibited the EGFR/AKT/NF-κB/IkB signalling pathway, which suppressed tumour proliferation. Additionally, compared to free DOX used during the trial, the researchers discovered that the TDE method of drug delivery minimized the harmful effects of the drug on the heart [157]. Cheng et al. piggybacked on this research to generate phase-fused synthetic nanovesicles carrying photothermal agents and immune adjuvants, which were formed by fusing TDEs carrying an abundance of CD47 with thermosensitive liposomes. By competing with CD47 on the tumour cell surface for the binding of CD47/SIRP on phagocytes, the nanovesicles overexpressing CD47 increased the phagocytosis rate of tumour cells by macrophages. In addition, nanovesicles that accumulated in tumour tissues played a role in immunogenic cell death via the in vitro action of the photothermal laser [158]. The dangerous side effects of chemotherapy medications have been decreased as a result of the development of EV-CAM-targeted drug delivery. Moreover, EV-CAMs show a wide range of applications since low-dose chemotherapy is possible because of their high absorption characteristics. However, many TDEs can promote tumour progression, counteracting the original aim of TDE drug delivery. EVs originating from specific cells types, including mesenchymal stem cell-derived EVs (e.g., NCT03608631), which are employed as substitutes for traditional drug delivery systems, share this issue [159]. In addition, CAMs are not the only factors involved in the biological activities induced by EVs; other components, such as other cytokines, ncRNAs, and lipids carried by EVs, play important functions [34]. Therefore, characterizing the contents of EVs and ensuring that they do not cause any undesirable inflammatory and cell-proliferative effects is of great importance [157]. In addition to laboratory trials, long-term clinical trials are needed to identify any adverse effects of EVs as therapeutic vehicles.

Oncology therapy and drug delivery involving engineered EV-CAMs have attracted much attention. However, a number of issues need to be addressed before their clinical application. The first issue that needs to be addressed involves increased large-scale production of EVs. Although the traditional differential ultracentrifugation separation method is inexpensive, it produces few EVs, and the EVs that are produced carry a risk of contamination with impurities [160]. Although density gradient ultracentrifugation separation can be used to obtain EVs with a high degree of purity, the procedure is laborious and time-consuming [161]. Other methods for producing large quantities of EVs, such as size exclusion chromatography [162], ultrafiltration [163], and immunoaffinity capture [164], also have drawbacks that need to be resolved. These drawbacks include the length of production time and expense, as well as the lack of guaranteed purity [161, 165, 166]). In conclusion, every method of separating EVs currently in use shows some disadvantages, and for research and development is needed to establish a method that is effective, affordable, and for which production quality can be controlled. The second challenge is the complex conditions needed to store EVs, as close attention is needed to ensure their stability during isolation, drug delivery, transportation and clinical application [167]. Further improvements are required because current storage conditions do not enable EV morphological or bioactivity maintenance for an extended period [168]. The third challenge involves the complex makeup of EVs. Since some EVs are immunogenic and pathogenic [169], it is important to fully evaluate their effects such as their potential toxicity and side effects and investigate their pharmacological and toxicological characteristics to create safe and useful engineered EVs.

#### EV-CAMs increase antitumour immunity

In addition to engineered EV-CAMs used for drug delivery, EV-CAMs have been evaluated in clinical trials with promising results because they induce antitumour immunity, palliating tumour growth, because of their immunomodulatory properties. The results of a clinical experiment with metastatic melanoma patients revealed that some patients exhibited expansion and recruitment of T cells in the tumour area as well as tumour regression after treatment with dendritic cell-derived extracellular vesicles (DEVs) loaded with MHC-II molecules [170]. A different clinical experiment with patients of non-small cell lung cancer demonstrated that MHC II levels on IFN-γ-DEV correlated with the activation of NK cells and prolonged progression-free survival [171]. With additional designs and further modification, DEV-induced immunity will be increased, enhancing its potential to be developed into an antitumour vaccine.

## Conclusions

CAMs can be loaded onto and released through EVs, which are crucial mediators of communication between tumour cells and tumour cells as well as between nontumour cells and tumour cells. By promoting pro-oncogenic effects (reprogramming of the TME and tumour cells) and impeding a body's defence against tumours (suppressing immunological destruction), CAM ^+^ EVs contribute to a TME that supports tumour development and proliferation. However, recent studies on the mechanisms underlying CAMs transport by EVs and their effects on tumours do not fully describe the full range of roles performed by EV-CAMs. However, we can still make some inferences based on the data thus far.

- 1. Since different organs and tissues express distinct ligands, differences in the types of CAMs transported by EVs from various cells of origin, EV in vivo distribution can be controlled by leveraging their preferentially targeted sites.
- 2. CAMs can not only facilitate the internalization of EVs to transmit information to receptor cells but also can facility communication between EVs and other cells by direct contact of CAMs on vesicle membrane with ligands on the plasma membrane.
- 3. EV-CAMs are extensively involved in the dynamic changes of the TME during tumour progression. They promote immunosuppressive functions of the TME and support tumour growth and metastasis through the promotion of angiogenesis, the EMT, ECM remodelling, the transmission of factors that promote cell invasion and proliferation and PMN formation. However, EV-CAMs can also trigger a certain antitumour response because of their significant contribution to antigen presentation and immune cell activation.
- 4. EVs can be produced as vehicles for targeted drug delivery based on CAMs due to their long-range delivery capacity, good biocompatibility, and minimal immunogenicity, offering a new method for the targeted treatment of malignancies. Because of their capacity to modulate immune function, they can also be employed as novel tools for the development of antitumour vaccines. EV-CAMs can also be used as biomarkers of liquid biopsy samples to provide clinical data for decision-making and prognostic assessment of tumour treatment due to their specific release in various cancers and stages of the disease.

The mechanism of action of EV-CAMs in tumours and their use in clinical diagnostics and treatment have been outlined in this article. However, many issues remain to be addressed. For example, extensive clinical trials are still needed to prove the validity of the therapeutic use of EV-CAMs. Additionally, the precise and effective separation of EV-CAMs for clinical use is challenging due to their heterogeneity. Furthermore, the cargos of EVs are complex, and it remains unclear whether other components exert regulatory effects on the CAMs carried by EVs and involved in cellular physiological processes. Fortunately, some assays performed for physical characterization (including nanoparticle tracking analysis, transmission electron microscopy, dynamic light scattering, tuneable resistive pulse sensing, etc.) and protein analyses (including western blotting, ELISA, mass spectrometry, small particle flow cytometry, etc.) in the field of EVs are being used [172]. These techniques provide opportunities for researchers to gain a better understanding of the mechanisms of action of EV-CAMs in tumours and improve strategies for cancer treatment development.

## Data Availability

Not applicable.

## Abbreviations

ALIX: ALG-2 interacting protein X
APC: Antigen-presenting cell
AR: Androgen receptor
ARF6: ADP ribosylation factor 6
CAFs: Cancer-associated fibroblasts
CAM: Cell adhesion molecule
CD44v5: CD44 variant isoform 5
CTCs: Circulating tumour cells
DCs: Dendritic cells
DEVs: Dendritic cell-derived extracellular vesicles
DOX: Doxorubicin
ECM: Extracellular matrix
EMMPRIN: Extracellular matrix metalloproteinase inducer
EMT: Epithelial-mesenchymal transition
eNOS: Endothelial nitric-oxide synthase
EOC: Epithelial ovarian cancer
ERK: Extracellular signal-regulated kinase
ESCRT: Endosomal sorting complex required for transport
ESEs: Early-sorting endosomes
EVs: Extracellular vesicles
Gal-3: Galectin-3
GBM: Glioblastoma
GPCR: G-protein coupled receptor
GSCs: Glioblastoma stem cells
HPMCs: Human peritoneal mesothelial cells
HSPG: Heparan sulfate proteoglycan
ICAM-1: Intercellular adhesion molecule 1
IL-10: Interleukin 10
ILVs: Intraluminal vesicles
ITG: Integrin
LFA-1: Lymphocyte function-associated antigen 1
LIMK: Lim kinase
LSEs: Late-sorting endosomes
MCs: Mesothelial cells
MDSCs: Myeloid-derived suppressor cells
MLCK: Myosin light-chain kinase
MMP: Matrix metalloproteinase
mTOR: Mammalian target of rapamycin
MVBs: Multivesicular bodies
ncRNAs: Non-coding RNAs
NEPrCa: Neuroendocrine PrCa
NPC: Nasopharyngeal carcinoma
PD-1: Programmed death protein-1
PD-L1: Programmed cell death-ligand 1
PLD2: Phospholipase D2
PMN: Premetastatic niche
PrCa: Prostate cancer
p-Smad3: Phosphorylated Smad3
ROCK: Rho kinase
RTK: Receptor tyrosine kinases
SHP2: Src homology 2 domain-containing tyrosine phosphatase 2
SNARE: Soluble NSF attachment protein receptor
TCR: T cell receptor
TDEs: Tumour-derived EVs
TGF-β: Transforming growth factor β
TGN: Trans-Golgi network
TME: Tumour microenvironment
TMZ: Temozolomide
TSPAN: Tetraspanin
